# Novel Approaches to Investigate One-Carbon Metabolism and Related B-Vitamins in Blood Pressure

**DOI:** 10.3390/nu8110720

**Published:** 2016-11-11

**Authors:** Amy McMahon, Helene McNulty, Catherine F. Hughes, J. J. Strain, Mary Ward

**Affiliations:** Northern Ireland Centre for Food and Health, Ulster University, Coleraine BT52 1SA, UK; McMahon-A13@email.ulster.ac.uk (A.M.); h.mcnulty@ulster.ac.uk (H.M.); c.hughes@ulster.ac.uk (C.F.H.); JJ.Strain@ulster.ac.uk (J.J.S.)

**Keywords:** riboflavin, methylenetetrahydrofolate reductase (MTHFR), hypertension, blood pressure, ABPM, DNA methylation

## Abstract

Hypertension, a major risk factor for heart disease and stroke, is the world’s leading cause of preventable, premature death. A common polymorphism (677C→T) in the gene encoding the folate metabolizing enzyme methylenetetrahydrofolate reductase (MTHFR) is associated with increased blood pressure, and there is accumulating evidence demonstrating that this phenotype can be modulated, specifically in individuals with the *MTHFR* 677TT genotype, by the B-vitamin riboflavin, an essential co-factor for MTHFR. The underlying mechanism that links this polymorphism, and the related gene-nutrient interaction, with hypertension is currently unknown. Previous research has shown that 5-methyltetrahydrofolate, the product of the reaction catalysed by MTHFR, appears to be a positive allosteric modulator of endothelial nitric oxide synthase (eNOS) and may thus increase the production of nitric oxide, a potent vasodilator. Blood pressure follows a circadian pattern, peaking shortly after wakening and falling during the night, a phenomenon known as ‘dipping’. Any deviation from this pattern, which can only be identified using ambulatory blood pressure monitoring (ABPM), has been associated with increased cardiovascular disease (CVD) risk. This review will consider the evidence linking this polymorphism and novel gene-nutrient interaction with hypertension and the potential mechanisms that might be involved. The role of ABPM in B-vitamin research and in nutrition research generally will also be reviewed.

## 1. Introduction

Hypertension, defined as a systolic/diastolic blood pressure (BP) of 140/90 mmHg or greater, is the world’s leading cause of preventable, premature death and is the most important risk factor for cardiovascular disease (CVD) and stroke. Hypertension is responsible for 7.5 million deaths worldwide and an estimated 12.8% of all deaths annually [1]. It is estimated that by 2025, 1.56 billion people worldwide will suffer with hypertension, and by 2030, costs to the global economy will soar to $274 billion [2,3,4]. Treating hypertension is highly effective in reducing CVD and mortality [5], with a decrease of as little as 2 mmHg in systolic BP reported to reduce CVD risk by 10% [6]. BP is normally treated using antihypertensive medication, but this is not always effective, and BP control rates can remain poor (even when three or more drugs are co-administered, i.e., polytherapy) [7,8]. Current guidelines to treat hypertension are aimed at achieving a BP of <140/90 mmHg [7]; however, recent evidence suggests that a more intensive treatment regime to reduce BP to as low as <120/80 may be significantly more beneficial, even when BP values fall within the normotensive range [8,9,10].

There are many well-known modifiable risk factors for hypertension, such as smoking, obesity, high salt intake and physical inactivity, and thus, addressing lifestyle factors is generally the first line of treatment recommended by medical practitioners to reduce BP [7]. Genome-wide association studies (GWAS) have however identified a number of areas in the genome related to the variability in BP, including a region near the *MTHFR* locus [11], a finding replicated by other GWAS [12,13,14]. Likewise, large meta-analyses of epidemiological studies have shown that adults with the homozygous variant (TT genotype) for the common *MTHFR* C677T polymorphism are at an increased risk of developing hypertension [15,16,17,18,19]. Riboflavin is required as a cofactor for MTHFR, and previous studies at this centre have shown that supplementation with riboflavin significantly reduces BP in adults with this genetic risk factor [20,21,22]. The mechanism by which riboflavin lowers BP in this genetically at-risk group is unknown; however, some mechanisms have been speculated, and these will be explored below [22,23]. 

All studies to date investigating this gene-nutrient interaction in hypertension have relied on clinic BP measurements. An alternative, more robust method of BP measurement is ambulatory blood pressure monitoring (ABPM), which can track the circadian pattern of BP, and it is reported to be a better predictor of mortality [24]. Despite the use of ABPM being first reported in the mid-1960s [25], it was not introduced into the relevant UK clinical guidelines to confirm the diagnosis of hypertension until 2011 [7]. 

## 2. One-Carbon Metabolism and Related B-Vitamins 

**Figure 1:**
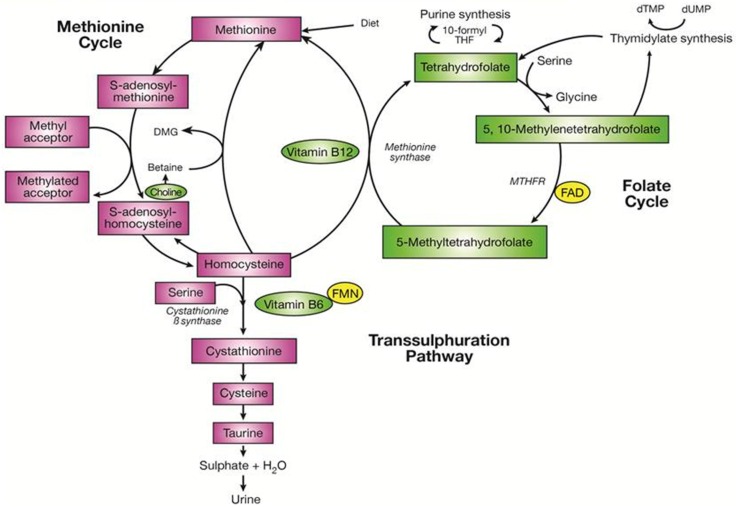
One-carbon metabolism pathway reproduced from Clarke et al. [31]. FAD, flavin adenine dinucleotide; FMN, flavin adenine dinucleotide.

In order to be biochemically active, folate needs to be in the fully reduced form as tetrahydrofolate (THF; Figure 1). Thus, folic acid, the synthetic vitamin form as found in supplements and fortified food, requires biological modification (via dihydrofolate reductase (DHFR)) to form THF [26]. This occurs in two consecutive NADPH-dependent reactions, to form dihydrofolate (DHF) and subsequently THF. The reduction of folic acid is, however, a slow process that is influenced by individual variation in DHFR activity [26]. It is possible therefore that exposure to high oral doses of folic acid may result in the appearance of unmetabolised folic acid in the circulation [27], which some have suggested may be associated with adverse health effects [28]. Once THF enters the folate cycle, it gains a methyl group from the conversion of serine to glycine in a vitamin B6-dependent (i.e., pyridoxal 5′-phosphate) reaction to form 5,10-methyleneTHF. Riboflavin also participates in one-carbon metabolism in its active co-factor forms flavin adenine dinucleotide (FAD) and flavin mononucleotide (FMN). Pyridoxine-phosphate oxidase requires FMN for the formation of the active form of vitamin B6, pyridoxal 5′-phosphate, from pyridoxine phosphate. MTHFR, which requires FAD as a co-factor, converts 5,10-methyleneTHF to 5-methylTHF which is subsequently converted to THF, in a reaction catalysed by methionine synthase, completing the cycle. The latter conversion also requires vitamin B12 (i.e., methylcobalamin) as a co-factor and simultaneously enables the remethylation of homocysteine to methionine and subsequently S-adenosylmethionine (SAM), the universal methyl donor, which is essential for a range of methylation processes, including DNA methylation. DNA methylation involves the addition of a methyl group to the DNA base cytosine, which can alter the transcription of the gene and potentially reduce enzyme production [29]. Thus, apart from folate, three other B-vitamins play essential roles in one-carbon metabolism, as they are required for the activity of the various enzymes within the folate cycle.

It is well established that the common *MTHFR* C677T polymorphism, which causes an amino acid change from alanine to valine in the protein, produces a thermolabile enzyme [30]. Individuals with the *MTHFR* 677TT genotype have 70% reduced activity of MTHFR in comparison to the *MTHFR* 677CC genotype, which in turn reduces the rate of 5-methylTHF production [30] and, potentially, SAM production in cells. The *MTHFR* 677TT genotype is thus associated with increased homocysteine concentration as the primary phenotype [30].

## 3. B-Vitamins, Cardiovascular Disease and Blood Pressure

Increased plasma homocysteine and/or poor status of the metabolically-related B-vitamins have been linked with an increased risk of CVD over many years, albeit the literature is somewhat controversial [32]. It is well established that intervention with folate, along with vitamin B12 and vitamin B6, can reduce plasma homocysteine concentrations (by about 3 µmol/L) as reported in a large meta-analysis of randomised controlled trials [33]. Of the B-vitamins investigated, folic acid has been shown to be most effective, even at doses as low as 0.2 mg/day when maintained for a long enough period of intervention (i.e., 24 weeks) [33,34]. Separate meta-analyses estimated that a reduction in homocysteine by 25% could reduce the risk of stroke by up to 24% and heart disease by 16% [35,36]. To subsequently establish a cause and effect relationship between elevated homocysteine and CVD outcomes, a number of randomised controlled trials with B-vitamins were conducted; however, despite significant homocysteine lowering, the majority failed to show a reduction in CVD risk in response to intervention with B-vitamins (as extensively reviewed elsewhere; [32,37]). Of note however, a secondary analysis of the Heart Outcomes Prevention Evaluation 2 trial (HOPE 2) and two subsequent meta-analyses demonstrated that interventions with folic acid and related B-vitamins could substantially reduce the risk of stroke [38,39,40]. This effect was generally confined to trials that were three years in length or greater (suggesting that trials of shorter duration may have underestimated or missed this benefit) and in trials involving participants with no previous history of stroke. However, the vast majority of the previous trials were secondary prevention trials in patients with overt CVD, and as such, participants would be less likely to respond to B-vitamin intervention. Furthermore, it has been reported that antiplatelet drugs, such as aspirin (commonly prescribed to patients after a CVD event) might mask any CVD risk reduction [41]. Of note, a recent primary prevention trial in 20,702 hypertensive Chinese adults reported that folic acid supplementation (0.8 mg/day), for a median duration of 4.5 years, reduced the risk of stroke in those with elevated cholesterol by as much as 31% [42]. The balance of evidence therefore suggests that any beneficial effects of B-vitamins would be most relevant in the prevention rather than the treatment of CVD.

Although most of the previous studies in relation to CVD were designed to examine homocysteine lowering in relation to CVD risk, some also considered the relationship of B-vitamins with BP. One of the largest studies, the VISP (Vitamin Intervention for Stroke Prevention) trial, which involved supplementing 3649 cerebral infarct patients with high or low doses of folic acid for two years, successfully reduced homocysteine, but showed no corresponding effect on BP [43]. Likewise, McMahon and colleagues also reported a significant reduction in homocysteine in response to supplementation with folic acid, B6 and B12 for two years, but no effect on BP [44]. One small study of just 130 individuals who were supplemented with high dose folic acid (5 mg/day) and B6 (250 mg/day) for two years, reported a reduction in homocysteine by 7.8 µmol/L and did achieve a corresponding lowering of systolic BP (−3.7 mmHg) [45]. Somewhat surprisingly, a further study of only 24 male smokers reported a smaller, albeit significant, reduction in homocysteine (2.6 µmol/L) and found a greater lowering of systolic BP (−8 mmHg SBP); however this study was conducted in adults who smoke, and it is well known that smoking increases homocysteine and BP [46,47]. If homocysteine was causally linked with increased BP, intervention studies to lower homocysteine would invariably show a corresponding BP response; however, this is not the case [32,37], suggesting there is no mechanistic association between homocysteine and BP. All of the aforementioned studies reported BP using the clinic BP monitor and have not considered the influence of *MTHFR* genotypes on BP response.

The *MTHFR* 677TT genotype has been independently associated with increased CVD risk [35,48,49,50,51]. This association is generally assumed to be owed to homocysteine, which is invariably found to be highest in those with the TT genotype and lowest in the CC genotype, with CT individuals having intermediate concentrations [32,35]. More recently, emerging evidence suggests that the excess genetic risk of CVD owed to this polymorphism may be through an independent association with BP. Furthermore, meta-analyses that have investigated the association between the TT genotype and CVD risk generally show a stronger relationship for stroke [49,51], than for heart disease [35,48,50,52]. Moreover, in studies focusing on the risk of hypertension (as opposed to CVD outcomes), the relationship is stronger again [15,16,17,18,19]. This is of importance as hypertension is a major risk factor for stroke [15,16,17,18,19]. Apart from hypertension in the general population [15,17,19], this polymorphism has also been linked with hypertension in pregnancy [16,18]. Given that hypertension is such a major risk factor for stroke, it is possible that the onset of stroke could be prevented or delayed by modulating the blood pressure phenotype in individuals with the *MTHFR* 677TT genotype. 

Among the studies investigating the association between MTHFR and BP, there are differences in the extent of risk linking this polymorphism with CVD including stroke [15,16,17,18,19]. Environmental factors affecting different populations, particularly B-vitamin status, could strongly influence the BP phenotype and thus stroke risk. Within these meta-analyses, the excess risk owed to the *MTHFR* 677TT genotype is found to be considerably higher in Asian populations for both CVD (OR 1.68, 95% CI 1.44–1.97) [51] and hypertension (OR 1.87, 95% CI 1.31–2.68) [16] and lower in North American populations. 

Leading on from earlier work, which demonstrated that supplementation with riboflavin could improve MTHFR activity in vivo (evident by decreasing plasma homocysteine concentrations) [53], researchers at this centre investigated the BP lowering effect of riboflavin in adults with the *MTHFR* 677TT genotype. In the first study, Horigan et al. [20] conducted a randomised controlled trial of riboflavin supplementation at 1.6 mg/day in premature CVD patients stratified for the *MTHFR* C677T polymorphism (*n* = 181). At baseline, BP control rates were poor in the TT genotype group, with 63% failing to reach the target BP of <140/90 mmHg on treatment with antihypertensive drugs. Systolic and diastolic BP were also significantly higher at baseline in the TT compared to the CC or CT genotype groups. In response to intervention with riboflavin (1.6 mg/day for 16 weeks), riboflavin biomarker status improved in all three genotypes in response to intervention (*p* < 0.001; as measured using the erythrocyte glutathione reductase activation coefficient). However, BP was significantly reduced in the TT genotype group only, with one third of patients in this group achieving a reduction in systolic BP by as much as 20 mmHg. When the *MTHFR* 677TT genotype participants from this first study were subsequently followed up four years later and assigned to further intervention in a cross-over design study (with the original treatments reversed), the BP lowering effects of riboflavin in the TT genotype group were replicated [21]. The BP lowering achieved in response to riboflavin occurred despite a major change in the UK clinical guidelines for the management of hypertension during the four-year follow-up period, resulting in a shift from monotherapy to polytherapy, thus additional medications being prescribed, and a change from β-blockers to angiotensin-converting enzyme (ACE) inhibitors as the drugs of first choice [7]. In a subsequent study, the genotype-specific BP lowering effects of riboflavin were also demonstrated in hypertensive adults without overt CVD [22]; albeit the extent of BP lowering was less pronounced (ranging from 5.6 to 13 mmHg systolic and 6–8 mmHg diastolic). It is not entirely clear why the extent of BP lowering was somewhat less in the last of these trials [22] compared to the previous ones [20,21], but recent evidence from our centre indicates that age and gender may strongly influence the BP phenotype [54]. To date, all studies investigating this gene-nutrient interaction in BP have intervened with riboflavin only. Given the close metabolic interplay between folate and riboflavin and the likelihood that enhancing MTHFR activity increases levels of 5-methylTHF, it is possible that supplementing with 5-methylTHF alone could have beneficial effects on BP in those with the *MTHFR* 677TT genotype, similar to that of riboflavin. Further research is required to investigate the role of 5-methylTHF in individuals with the *MTHFR* 677TT genotype. In any case, these findings have important implications for BP management for subpopulations worldwide given the high frequency of the *MTHFR* 677TT genotype (on average 10%, but as high as 32% in Mexico) [55]. Although biomarker status of riboflavin is rarely measured in populations, deficient and low status appears to commonly occur, especially in countries where intakes of riboflavin-rich food are low [56]. Further studies are clearly required to investigate this phenotype in younger adults in both genders and to consider the potential for preventing the development of hypertension through optimising riboflavin status. Of note, to date, all studies investigating the *MTHFR* C677T polymorphism and BP have utilized clinic BP. Ambulatory blood pressure monitoring (ABPM) offers an alternative method, which measures BP over a 24-h period and will be discussed later.

## 4. Biological Mechanisms Linking Blood Pressure with One-Carbon Metabolism

When the *MTHFR* C677T polymorphism was first described, Frosst et al. [30] reported that there is a 70% reduced activity in the mutated enzyme as measured in human lymphocytes. Further evidence from in vitro studies in *Escherichia coli* demonstrated that the mutated MTHFR enzyme loses its FAD cofactor at a much greater rate than the wild-type enzyme, reducing the overall functioning of the enzyme [57]. This was supported by a subsequent in vitro study in recombinant human MTHFR, which reported that in low folate compared to optimal folate conditions, the loss of FAD co-factor from the mutated enzyme was exacerbated [58]. As discussed previously, the FAD-dependent enzyme MTHFR is essential for the production of 5-methylTHF within the one-carbon metabolism cycle. Bagely and Selhub [59] observed decreased concentrations of 5-methylTHF and an accumulation of 10-formylTHF in red blood cells of individuals with the TT genotype, indicative of altered red blood cell folate distribution as a result of decreased MTHFR activity. It has also been postulated that the mutated enzyme has an altered active site, therefore reducing the efficacy of the binding of FAD [60]. It is not known how enzyme activity is enhanced by supplementation with riboflavin [53], but it is possible that this is achieved through stabilization of the variant enzyme and potentially increasing the enzyme-substrate binding, thereby improving its function [23]. 

The potential mechanism underlying the role of MTHFR in hypertension is currently unknown, but may involve endothelial nitric oxide synthase (eNOS). In one study involving patients undergoing cardiac bypass surgery, researchers found that vascular tissue concentrations of 5-methylTHF were positively associated with endothelial function (regardless of *MTHFR* C677T genotype) via the production of nitric oxide, a potent vasodilator [61]. A subsequent study from the same researchers reported that vascular 5-methylTHF is an important regulator of eNOS coupling and nitric oxide bioavailability [62]. It is possible therefore that supplementation with riboflavin (i.e., the MTHFR cofactor) in individuals with the *MTHFR* 677TT genotype could increase MTHFR enzyme activity in vivo, resulting in an increased cellular production of 5-methylTHF and, thus, lower BP by promoting vasodilation [22,23,61]. 

A further, and possibly complementary, mechanism to explain this novel gene-nutrient interaction in hypertension may involve epigenetic modification. Epigenetics can change the expression of a gene without altering the underlying DNA sequence by histone modification, RNA interference or DNA methylation [29]. The majority of research into epigenetic modifications is in relation to DNA methylation. Folate, owing to its importance in one-carbon metabolism and the production of SAM, plays a key role in modulating DNA methylation levels, of relevance to diseases, such as cancer [63], and folic acid supplementation during pregnancy has also been reported to alter DNA methylation in the offspring [64]. Furthermore, animal studies have shown that in folate-deficient mice, aberrant DNA methylation occurs at the promoter regions of the *MTHFR* gene, resulting in alterations in gene expression [65]. In support of these findings, the same researchers have found increased methylation in the promoter region of the *MTHFR* gene in paediatric cancerous tissues [65]. One study that investigated the *MTHFR* C677T polymorphism and folate status in relation to DNA methylation found that the TT genotype combined with low folate status was associated with low genomic DNA methylation levels [66]. A reduction in genomic DNA methylation levels could lead to altered gene expression, and optimizing folate status in these individuals could counteract this effect.

One meta-analysis of observational studies examined the relationship between *MTHFR* single nucleotide polymorphisms and genomic DNA methylation. Of the 16 studies included, 10 studies examined the role of the *MTHFR* C677T polymorphism on global DNA methylation and found no significant association. It is important to note however that the studies included in this meta-analysis were designed to examine a number of diseases and were not focused on hypertension alone [67]. Elsewhere, long interspersed element-1 (LINE-1) methylation (an indication of global methylation) has been reported to be significantly lower in individuals with the *MTHFR* 677TT genotype compared to those with the CC genotype [68]. Furthermore, adults with the TT genotype compared to the CC genotype had diminished genomic DNA methylation, and this correlated with folate status [66]. There is also some evidence linking pre-eclampsia (characterised by proteinuria and hypertension) with *MTHFR* gene hypermethylation [69]. However, DNA methylation at *MTHFR* has not been examined to any great extent in relation to hypertension generally. Aberrant LINE-1 and gene-specific DNA methylation profiles have been reported to improve the prediction accuracy of the occurrence of myocardial infarction, when other well-known risk factors for hypertension are taken into account [70]. One randomised controlled trial found that B-vitamin supplementation (folic acid, vitamins B6 and B12) in combination with vitamin D and calcium, increased LINE-1 DNA methylation, but this study did not account for different *MTHFR* genotypes [71]. 

As MTHFR is required for normal folate recycling and thus the generation of SAM, stabilization of the mutated enzyme by riboflavin supplementation may overcome any possible aberrant DNA methylation owing to this polymorphism. In any case, further studies are required to investigate DNA methylation in relation to MTHFR-mediated blood pressure and to ascertain whether DNA methylation is altered by riboflavin supplementation in the TT genotype group.

## 5. Ambulatory Blood Pressure Monitoring

BP can be measured in a number of ways in clinical and research settings. The most common and convenient method of BP measurement of patients in the clinic is with a standard sphygmomanometer; however, as a result of BP variability, a one-off clinic BP reading may be a poor representation of an individual’s true BP [72]. Out-of-office BP measurement, such as home BP monitoring (HBPM) and ABPM, is recognised as a more accurate measure of BP, as it is not influenced by the clinical environment. ABPM is considered to be more superior to HBPM, as it is a non-invasive and a robust measure of BP owing to its ability to record BP over a defined period of time, usually 24-h. This continuous monitoring accounts for the daily variability in BP and, as such, is regarded as the gold standard in BP monitoring [73]. The 24-h window of BP monitoring can be further sub-divided into: the white coat window, daytime window, nighttime window and morning increase in BP [74], with each period being independently associated with CVD risk (as will be discussed further below). Despite being first reported in the early 1960s [25], ABPM has only recently been introduced into UK clinical guidelines as a method of confirming a diagnosis of hypertension [7]. The current guidelines recommend that hypertensive patients, once diagnosed, should be monitored on an annual basis using ABPM.

There are a number of important differences between clinic BP and ABPM, as summarised in Table 1, which are discussed in detail below. The most important advantage of ABPM is that it provides a minimum of 14-day time systolic and diastolic BP readings, allowing a more accurate diagnosis of hypertension to be made [75]. These multiple readings improve the prediction of CVD risk and stroke, as clinic BP only provides a ‘snap-shot’ of the BP [76,77]. Of note, different cut-off values are used for diagnosing hypertension, depending on whether clinic BP or ABPM is used. Clinical guidelines in the UK define hypertension as a clinic BP reading (systolic/diastolic) of ≥140/90 mmHg, whereas using ABPM, the corresponding cut-off values are ≥135/85 mmHg [7]. 

Along with providing multiple measurements of systolic and diastolic BP, continuous monitoring by ABPM provides a wealth of additional relevant information including mean arterial pressure, pulse pressure and heart rate, each of which are independently associated with CVD risk [78]. Although encompassing aspects of systolic and diastolic BP, the relevance of mean arterial pressure (i.e., defined as ⅔ diastolic BP + ⅓ systolic BP), is often underestimated as an independent risk factor for hypertension. In one large study of 11,150 adults, mean arterial pressure was linked with an increased risk of CVD, and this association was strongest in younger males (relative risk 2.52 95% CI 1.87–3.40 compared to 1.43 95% CI 1.07–1.92 in older males) [78]. The relevance of pulse pressure (i.e., defined as the difference between systolic BP and diastolic BP) as a predictor of CVD risk has been more extensively investigated, as it is an indicator of large arterial stiffness and isolated systolic hypertension. As people age, systolic BP increases, and diastolic BP remains steady or may even decrease, ultimately resulting in an increased pulse pressure, with estimates that each 10 mmHg increase in pulse pressure is associated with a 22%–35% increased risk of fatal stroke [79,80] and a 35% increased risk of cardiac events [80]. Thus, increased pulse pressure is now recognised by the European Society of Hypertension as an independent risk factor for cardiovascular events and is distinct from the risk associated with the increase in systolic BP with ageing [81]. Heart rate is another independent risk factor, which can be measured using ABPM. Increased heart rate has been associated with increasing BP independently of age and sex [82,83]. An increase in heart rate of 10 beats/min is associated with increased risk of cardiac death by as much as 20% and is also related to the severity of atherosclerosis at all ages [83,84]. In contrast, pulse pressure, which increases as people age, is more important for determining CVD risk in the later stages in life. Targeting heart rate could therefore potentially offer a therapeutic option when treating hypertension; however, results to date from trials are inconsistent [85].

### 5.1. White-Coat Hypertension

ABPM also offers the advantage of enabling the identification of a phenomenon first described by Pickering et al. in 1988, named white-coat hypertension (WCH). This occurs when a patient has BP readings in the hypertensive range as measured in the clinic, but normal BP when monitored throughout the day [86]. WCH is thought to affect up to 30% of the general population [87] and can only be detected by the use of HBPM or ABPM. The main advantage of identifying WCH is to ensure antihypertensive medication is only prescribed to individuals who require it. Furthermore, dietary and lifestyle changes may offer greater benefit to patients with WCH, at least initially [88].

Although earlier studies investigating WCH did not link it with additional CVD risk [89], more recent research has associated WCH with an increased risk of CVD [90,91]. A recent meta-analysis of 29,100 participants reported that patients with WCH (compared to normotensive controls) were at an increased risk of incidence of cardiovascular events (OR 1.73, 95% CI 1.27–2.36) and CVD mortality (OR 2.79, 95% CI 1.62–4.80), although not all-cause mortality or stroke [92]. Two recent studies have also shown that WCH is associated with subclinical arterial wall damage, including thickening of the arterial wall [93,94]. Whilst the association between WCH and CVD risk is not fully understood, it might be due to BP variability, which is associated with increased risk of CVD events and mortality [95]. Randomised controlled trials are required to determine if reducing the BP in WCH patients could reduce CVD risk. Aside from WCH, ABPM also has the ability to detect masked hypertension (i.e., normotensive clinic BP, but hypertensive ABPM) and resistant hypertension (i.e., difficult to treat hypertension). Masked hypertension is generally undetected owing to the appearance of normal BP in the clinic and is therefore associated with a higher risk of cardiovascular events as it remains untreated [90]. In relation to resistant hypertension, ABPM can identify patients that have true resistant hypertension (unresponsive to three or more antihypertensive medications), and is reviewed extensively elsewhere [96,97].

### 5.2. Circadian Pattern of Blood Pressure

The benefit of being able to trace the circadian pattern of BP is that it enables the identification of those who do not follow the expected pattern, in particular, where BP rises sharply in the morning and falls at night, while sleeping (a phenomenon known as ‘dipping’). When first reported, individual patients were categorised into either a dipping or non-dipping pattern [98]. The non-dipping pattern has been independently associated with increased risk of CVD in many studies [99,100,101,102,103,104] and has also been associated with increased CVD risk in adults without hypertension [105]. It is now more common to further divide non-dippers into three groups; thus, four groups in total referred to as: dippers (considered to have a normal dipping pattern; ≥10%–20% fall at night), extreme dippers (≥20%), non-dippers (≥0%–10%) or risers (increase in BP), depending on how much their BP falls at night in comparison to the average daytime BP. 

Meta-analyses of studies that have investigated the circadian pattern of BP in relation to CVD events are summarised in Table 2. Salles et al. conducted a large meta-analysis in over three continents to investigate the effect of various dipping patterns on cardiovascular events and stroke [106]. Compared to the normal dipping pattern, individuals with any other dipping pattern at night were found to be at an increased risk of cardiovascular events (OR 1.40, 95% CI 1.20–1.63) and in particular stroke (OR 1.43, 95% CI 1.15–1.77). When the non-dipping pattern was further subdivided, a rising pattern (increase in BP at night), in comparison to the normal dipping pattern, increased the risk of CVD by 79% and stroke by 89%. An increase in the morning surge of BP has also been associated with increased risk of coronary events [107], suggesting that varying circadian patterns of BP (daytime or nighttime) can increase the risk of CVD. Other large meta-analyses investigating the variation in circadian pattern in different populations support this finding, with estimates of excess CVD risk owing to atypical dipping ranging from 15% to 49% [102,108,109].

The mechanism for deviation in the circadian dipping pattern is unknown. Speculated mechanisms include increased sympathetic nervous system activity [110,111], decreased baroreceptor reflex sensitivity [112], increased arterial stiffness [113] and endothelial dysfunction [114]. Ageing is also known to play a role in the reduced decline in BP at night as there is an increase in the activity of the sympathetic nervous system and a decrease in baroreceptor reflex sensitivity. Poor quality of sleep has also been associated with increased BP readings at night and therefore could provide inaccurate reporting of dipping status [115]. Measures used to minimize the risk of inaccurate readings include ensuring that the right size of BP cuff is used, that the patient is fully informed of the procedure and a diary of events is kept (including any disturbed sleep). Although the reproducibility of nighttime dipping pattern (based on ABPM carried out on different days) has been questioned by some [116], Keren et al. reported that 71% of individuals reproduced the same dipping pattern when two 24-h readings were taken within 14 weeks [117].

In the clinical setting, ABPM can also be used to monitor the BP response to antihypertensive medication and to detect under- or over-treatment. Some patients present with resistant hypertension (the ineffective response to three or more antihypertensive medications) and chronotherapy can be introduced to potentially overcome this. Chronotherapy, in relation to hypertension treatment, is the administration of medication at a certain time of day, in line with the peaks in BP (as measured by ABPM) to improve the patient’s response to treatment [118]. If the non-dipper or riser BP pattern at night is present, ingestion of medication at night, rather than in the morning, may be more beneficial [118,119]. One study reported that changing the time of administrating medication from morning to night significantly reduced BP (*p* < 0.001) in 250 hypertensive adults [118]. 

In summary, given these clear advantages, ABPM should be used in the clinical setting to diagnose hypertension accurately, to identify those at risk through abnormal circadian patterns and to help identify the optimum time for taking antihypertensive medication to maximise its effect. Further research is required to understand the effects of WCH and the potential effects of lowering BP in this at-risk group. 

### 5.3. Nutrition, B-Vitamins and ABPM

As mentioned above, diet and lifestyle factors are usually suggested as the first line of treatment for the management of hypertension before pharmacological intervention is commenced, unless other CVD risk factors have been noted [7]. Many studies have investigated BP response to nutrition intervention; however, few studies have used ABPM to measure BP response. Those studies that have generally tend to report mean systolic BP and diastolic BP only, with many failing to consider the wealth of additional information that ABPM can contribute. The case for the use of ABPM in nutrition interventions is strong as ABPM has been reported to rule out the placebo effect after the first initial hours of recording BP, providing a more robust measure of potential BP reduction in intervention trials [120,121].

The relationship between obesity and BP as measured by ABPM has been investigated, confirming that BP increases with increasing BMI and showing that the non-dipping pattern is more prevalent in those with a BMI in the obese or severely obese range [122,123]. Micronutrients have also been investigated in relation to BP, and vitamin D deficiency has been associated with increased BP and with the non-dipping BP at night [124,125]. Other studies have reported that higher concentrations of serum calcium, phosphate and parathyroid hormone are associated with the non-dipping pattern in hypertensive patients without renal disease [126]. The majority of other studies failed to report the dipping pattern, mean arterial pressure, heart rate and/or pulse pressure despite the use of ABPM [127,128,129,130,131,132,133,134,135,136,137,138,139].

A limited number of randomised controlled trials have examined ABPM response to nutrition interventions, with somewhat inconsistent findings (Table 3). One of the first randomised controlled trials to investigate the ABPM response to a nutrition intervention (and the most comprehensive study to date) involved the Dietary Approaches to Stop Hypertension (DASH) trial [127]. The DASH diet incorporates low fat dairy products along with high intakes of fruit and vegetables and is characterised by being rich in protein and fibre, as well as potassium, magnesium and calcium, and low in saturated fat and cholesterol [140]. In an eight-week study investigating the ABPM response to the DASH diet versus a control diet or a diet rich in fruits and vegetables, the DASH diet was shown to result in the greatest BP lowering, achieving a mean systolic BP lowering of 4.5 mmHg [127]. Two additional DASH studies, also reporting ABPM, confirmed these findings and reported a lowering of systolic BP of between 9.5 and 15 mmHg in response to intervention [128,129]. The greatest lowering achieved (a mean reduction in systolic BP of 15 mmHg) reported by Paula et al. is likely to be explained by the inclusion of an exercise component within this intervention [129]. 

### 5.4. Blood Pressure Lowering by B-Vitamins as Measured by ABPM

Few studies to date have investigated the BP response to B-vitamin intervention specifically using ABPM. One small study of 30 postmenopausal women reported that high-dose folate supplementation (in the form of 5-methylTHF, the product of the reaction catalysed by MTHFR), significantly decreased nighttime systolic BP (−4.48 ± 1.8 mmHg; *p* = 0.029), diastolic BP (−5.33 ± 1.3 mmHg; *p* < 0.001) and mean arterial pressure (−5.10 ± 1.1 mmHg; *p* = 0.005), with no effect of daytime BP [141,142]. ABPM has been used in one study that investigated the effect of folic acid supplementation on BP by the *MTHFR* C677T genotype [143]. Although ABPM was reported, the study was confined to hypertensive males, and ABPM was not reported as a primary outcome measure. No BP lowering effect was noted in this study, although a significant decrease in brachial pulse pressure (PP) (4.7 ± 1.6 mmHg; *p* < 0.05) was observed across *MTHFR* genotype groups in response to intervention [143]. To date, the randomised controlled trials investigating this polymorphism in relation to BP have focused on folic acid/folate supplementation, and no previous ABPM studies have considered the BP lowering effects of riboflavin, arguably much more important, given its role in stabilizing MTHFR. 

## 6. Conclusions and Future Work

Evidence is accumulating to support the role of the *MTHFR* C677T polymorphism in hypertension and indicates that the BP phenotype may be much more relevant to CVD than the metabolite homocysteine. Riboflavin is an important modulator of the BP phenotype in individuals with the *MTHFR* 677TT genotype, but further work is required to investigate the influence of age and gender on this gene-nutrient interaction in BP. The biological mechanism by which riboflavin lowers BP in individuals with the variant *MTHFR* 677TT genotype is unknown; however, aberrant DNA methylation should be considered, along with other postulated mechanisms. Studies to date investigating the BP-lowering effect of riboflavin on genetically at-risk individuals and in nutrition studies generally have utilized clinic BP, but ABPM is now accepted as being a more robust measure of BP and a better indicator of cardiovascular health and disease risk. Its use in this area and in nutrition research generally should be considered. Given the global burden of hypertension, further research is required to understand the role of the *MTHFR* C677T polymorphism in BP, the modulating effect of riboflavin and the implications of this novel gene-nutrient interaction for the diagnosis and management of hypertension in different populations.

## Figures and Tables

**Table 1:** Clinic BP vs. ABPM.

CLINIC BP	ABPM
Defines hypertension as ≥140/90 mmHg *	Defines hypertension as ≥135/85 mmHg * (daytime average)
Convenient, quick, non-invasive method to measure blood pressure One off measurement of: Systolic and Diastolic BP Pulse Rate	Robust measure of BP over a 24-h period Average 24-h/daytime/night-time measurement of: Systolic BP and Diastolic BP Mean Arterial Pressure Pulse Pressure Heart Rate
	Identifies white-coat/masked/resistant hypertension; A superior predictor of mortality; Provides information on: Circadian pattern Morning surge % time in hypertensive state over 24-h Night-time dipping pattern

* Blood pressure is recorded as systolic BP/diastolic BP.

**Table 2:** Meta-analyses of circadian pattern abnormalities and cardiovascular events.

Author	Sample Size (*n*)	Populations	ABPM 24-h Profile	Odds Ratio (95% CI) for Cardiovascular Events
Salles et al. [106]	17,312 hypertensives	South America, Europe and Asia	Non-dippers vs. dippers *	1.40 (1.20–1.63)
Xie et al. [108]	14,133 hypertensives	Europe, South America and Asia	Morning surge	1.24 (0.60–2.53)
Hansen et al. [109]	23,856 hypertensives	Europe, South America and Asia	Non-dippers vs. dippers *	1.25 (1.02–1.52)
	9641 general population			1.15 (1.00–1.33)
Fagard et al. [102]	3468 hypertensives	Europe	Reverse dippers vs. other dipping patterns	1.49 (1.17–1.91)

Definition of dipping patterns (i.e., blood pressure fall at night comparted to daytime average): dippers ≥10%–20% fall in BP; non-dippers ≥0%–10% fall in BP; extreme dippers ≥20% fall in BP; reverse dippers, rise in BP; morning surge in BP is the increase in BP shortly after awakening. * These studies compare the normal dipping patterns of ≥10%–20% (dippers) and all other dipping patterns combined, collectively referred to as ‘non-dippers’.

**Table 3:** Nutritional studies that report blood pressure response using ABPM in randomised controlled trials.

Author	Population	Sample Size	Average Systolic BP Change (mmHg)	Duration of Intervention	ABPM Parameters Reported			
					Dipping Status	Mean Arterial Pressure	Heart Rate	Pulse Pressure
**DASH Diet Interventions**								
Moore et al. [127]	USA	354	−4.5 ^≠^	8 weeks	Yes	No	No	No
Miller et al. [128]	USA	44	−9.5 ^≠^	9 weeks	No	No	No	No
Paula et al. * [129]	Brazil	40	−15.0 ^†^	4 weeks	No	No	Yes	No
**Dairy Product Interventions**								
Drouin-Chartier et al. [130]	Canada	89	−2.0 ^†^	4 weeks	No	No	No	No
Machin et al. [131]	USA	49	−8.0 ^≠^	4 weeks	No	No	No	Yes
**Beetroot Interventions**								
Hobbs et al. [132]	Europe	24	-	Acute	No	Yes	Yes	Yes
Jajja et al. [133]	Europe	24	-	3 weeks	No	No	Yes	No
Kapil et al. [134]	Europe	68	−7.7 ^≠^	4 weeks	No	No	Yes	No
Coles et al. [135]	Australia	30	−4.6 ^†^	Acute	No	No	Yes	Yes
**Vitamin D Intervention**								
Larsen et al. [136]	Europe	112	−3 ^≠^	20 weeks	Yes	No	Yes	No
Pilz et al. [137]	Europe	200	-	8 weeks	No	No	No	No
**Other**								
Brull et al. [138]	Europe	70	−3.6 ^≠^	6 weeks	Yes	Yes	Yes	No
Sauder et al. * [139]	USA	30	−3.5 ^≠^	4 weeks	Yes	No	Yes	No

* Denotes studies in diabetic patients; acute studies refer to one-off ingestions of beetroot product and effects monitored over 6–24 h. ^≠^ Systolic BP response over 24h; ^†^ systolic BP response during daytime. Brull et al. supplemented adults with quercetin-rich onion skin extract; Sauder et al. supplemented adults with pistachio nuts.
